# Electrochromic nanopixels with optical duality for optical encryption applications

**DOI:** 10.1515/nanoph-2023-0737

**Published:** 2024-01-12

**Authors:** Joo Hwan Ko, Ji-Eun Yeo, Hyo Eun Jeong, Dong Eun Yoo, Dong Wook Lee, Yeon-Wha Oh, Sanghee Jung, Il-Seok Kang, Hyeon-Ho Jeong, Young Min Song

**Affiliations:** School of Electrical Engineering and Computer Science, Gwangju Institute of Science and Technology (GIST), Cheomdangwagi-ro 123, Buk-gu, Gwangju 61005, Republic of Korea; National Nanofab Center, Korea Advanced Institute of Science and Technology, 291 Daehak-ro, Yuseong-gu, Daejeon 34141, Republic of Korea; School of Electrical Engineering and Computer Science and Department of Semiconductor Engineering, Gwangju Institute of Science and Technology, Gwangju 61005, Republic of Korea; School of Electrical Engineering and Computer Science, Department of Semiconductor Engineering, and Artificial Intelligence (AI) Graduate School, Gwangju Institute of Science and Technology (GIST), Cheomdangwagi-ro 123, Buk-gu, Gwangju 61005, Republic of Korea

**Keywords:** electrically tunable, nanowire, waveguide, structural color, optical information

## Abstract

Advances in nanophotonics have created numerous pathways for light–matter interactions in nanometer scale, enriched by physical and chemical mechanisms. Over the avenue, electrically tunable photonic response is highly desired for optical encryption, optical switch, and structural color display. However, the perceived obstacle, which lies in the energy-efficient tuning mechanism and/or its weak light–matter interaction, is treated as a barrier. Here, we introduce electrochromic nanopixels made of hybrid nanowires integrated with polyaniline (PANI). The device shows optical duality between two resonators: (i) surface plasmon polariton (SPP)-induced waveguide (wavelength-selective absorber) and (ii) ultrathin resonator (broadband absorber). With switching effect of between resonant modes, we achieve enhanced chromatic variation spanning from red to green and blue while operating at a sub-1-volt level, ensuring compatibility with the CMOS voltage range. This modulation is achieved by improving the light–matter interaction, effectively harnessing the intrinsic optical property transition of PANI from lossy to dielectric in response to the redox states. In our experimental approach, we successfully scaled up device fabrication to an 8-inch wafer, tailoring the nanowire array to different dimensions for optical information encryption. Demonstrating distinct chromaticity modulation, we achieve optical encryption of multiple data bits, up to 8 bits per unit cell. By capitalizing on the remarkable sensitivity to the angular dependence of the waveguiding mode, we further enhance the information capacity to an impressive 10 bits per unit cell.

## 1 Introduction

Reconfigurable optical modulators play a pivotal role in advancing various components, including optical memories, optical interconnects, and photonic switches [1]–[4]. Electrically controllable photonic device within the visible spectral region is critical for applications such as optical data storage, optical clocks, 3D displays, and augmented/virtual reality (AR/VR), as it would enable concurrent control of multiple optical modulators at the device level [5]–[9]. One of the strong candidates for optical modulation is the electro-optic effect, which induces changes in the refractive index in response to an applied electric field [10], [11]. Despite its excellent optical properties and compatibility with CMOS fabrication, it still faces challenges, primarily related to the high operational voltage and relatively small variations in the complex refractive index [12], [13]. An alternative and highly promising approach involves the use of active materials capable of modulating carrier densities, allowing direct control of plasmonic effects and the transformation of material optical constants [14]–[16]. Extensive exploration has been conducted to identify potential candidates for active materials, encompassing conducting oxides [15], phase change materials [17], and 2D materials [18]. Nevertheless, the challenge of meeting the criteria for efficient reconfiguration with energy-efficient operation remains [19]. These crucial factors include attaining a substantial change in the complex refractive index, preferably at low operational voltages.

Conducting polymers stand out among numerous active materials due to their appealing features, including high stability, affordability, and ease of synthesis [20], [21]. In recent years, polyaniline (PANI) has opened up opportunities for reconfigurable photonics due to its significant modulation in the complex refractive index within the visible wavelength range and energy efficiency operation [22]–[25]. PANI exhibits substantial alterations in its refractive index when subjected to electrochemical switching between its oxidized and reduced forms, especially within a voltage range of 1 V. In its oxidized state, PANI exhibits pronounced absorption, while in the reduced state, it virtually exhibits no absorption within the visible and near-infrared wavelength ranges [26]–[28]. While PANI holds promise as an active material, its inherently low refractive index results in inadequate light–matter interaction [18]. To address this limitation, focusing on PANI’s optical properties, which enable a complete transition from lossy to dielectric behavior, is essential. Consequently, designing the photonic structure to exhibit a sensitive response associated with the presence of the extinction coefficient becomes a prerequisite. In this context, the waveguiding mode based on surface plasmon polaritons (SPPs) proves highly sensitive to the surrounding atmosphere concerning the background complex refractive index [29]. Furthermore, optimizing the variation level in chromatic responses can be achieved by leveraging the multiple resonances [30].

Here, we propose an efficient photonic–plasmonic system that utilizes the properties of SPPs in metal nanowires integrated with a low-loss active material (PANI), transitioning from lossy to dielectric behavior. To expand the resonance frequencies and shift them within the visible wavelength range, we configured optical duality by incorporating the SPP-based nanowire into a broadband absorber. Our proposed resonator comprises two fundamental components: (i) a selective absorber incorporating an SPP-based waveguide and (ii) a broadband absorber based on an ultra-thin planar resonator. The Au encapsulation covering the Si nanowire induces the narrowband plasmonic resonance. Therefore, in the reduced state of PANI, this mode sustains strong absorption. However, when PANI is oxidized, the waveguided resonance weakens. Meanwhile, the increased extinction coefficient induces a nontrivial phase shift in the planar ultrathin resonator (i.e., Au/PANI), leading to strong and broad absorption [31], [32]. Leveraging the resonant mode transition effect, the electrochromic nanopixel (ENP) demonstrates a broadened chromatic range that spans across red, green, and blue colors, all while operating on a sub-1-volt input voltage, which is in line with the conventional CMOS voltage standard of 3.3 V [13]. Finally, this work extends to the practical demonstration of PANI-based devices: optical information encryption. Electrically programmable modulator cells with diverse structural parameters, serving as optically readable color filters, are demonstrated in an array. These cells are capable of generating 8 bits of optical information per unit cell. Moreover, by capitalizing on the high sensitivity to the angle dependency of the waveguiding mode, the information capacity is further increased to 10 bits per unit cell.

## 2 Results and discussion

### 2.1 Electrochromic nanopixels with hybrid nanowire array

**Figure 1:**
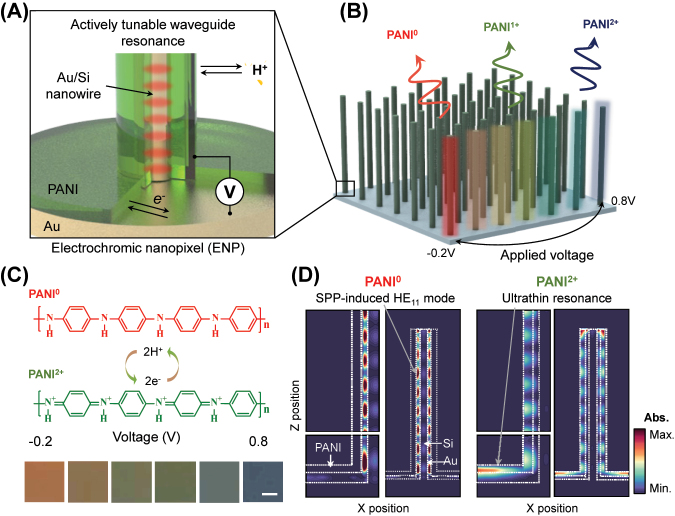
Electrochemically tunable nanowire array based on polyaniline (PANI). (A) Schematic of an electrochromic nanowire (ENP), which consists of polyaniline (PANI)/Au coating on Si nanowire. Each charge carrier (proton and electron) is exchanged. (B) Conceptual graphic image of ENP array with color change corresponding to the modulation of applied voltage. (C) Redox reaction of PANI, showing intermediate states (PANI^0^, fully reduced; PANI^1+^, half oxidized; PANI^2+^, fully oxidized). Scale bar is 1 mm. (D) Absorption profile of ENP corresponding to different redox states (PANI^0^ and PANI^2+^) at *λ* = 670 nm.

Color dynamics based on a waveguiding resonance operate by changing the refractive index of the medium surrounding silicon nanowires (Si NWs). As depicted in Figure 1A, our suggested structure can be explained by separating in two parts: resonator 1 for selective absorption (Au/PANI coated Si nanowire) and resonator 2 as a broadband absorber (Au/PANI planar resonator). In case of resonator 1, the Au/PANI coated Si nanowire generates narrowband plasmonic resonance, resulting in strong absorption as it guides incident light within Au tube filled with Si [29]. However, when PANI is oxidized, its lossy property results in the weakening of nanowire-based plasmonic resonance. In the case of resonator 2 (i.e., Au/PANI planar resonator), the lossy nature of PANI^2+^ leads to the ultrathin resonance with a nontrivial phase shift on the Au mirror [33]–[35]. Among a variety of conducting polymers, PANI is particularly attractive because of its high stability, low cost, and facile synthesis [36]. These hybrid silicon nanowires are arranged periodically, as depicted in the device schematic in Figure 1B, and exhibit tunable colors, modulated by applying electrochemical potentials. Crucially, the potential difference below 1 V leads to the full change in the redox states of the PANI layer, formed through the whole surfaces of the Si NWs (Figure 1C). When the PANI is electrochemically switched from its reduced (PANI^0^, top) to oxidized form (PANI^2+^, bottom), it undergoes significant changes in its complex refractive index, including the real and imaginary parts [26], [27]. Depending on the level of the applied voltage, an intermediate redox state can be also achieved, including the half-oxidized state (PANI^1+^), enabling the multicolor states from the single nanopixel. Notably, within the sub-1-volt range, the PANI^2+^ state exhibits strong absorbance, whereas the PANI^0^ state demonstrates minimal absorbance in the visible and near-infrared wavelength ranges [5], [28]. Through the described resonance mode switching and the resulting complementary shift in resonant wavelengths, chromaticity is significantly improved. The photographic image illustrates distinct color variations in ENPs, spanning across the visible colors, including red, green, and blue, even within the applied voltage range from −0.2 V to 0.8 V (Figure 1C; bottom).

The resonant mode switching effect can be observed in Figure 1D, which illustrates the absorption profile of a single unit cell of ENPs corresponding to the redox state of PANI. When PANI is in the PANI^0^ state, its optical transparency within the visible wavelength range allows incident light to be captured by the Au shell, while the planar resonator (substrate) shows no absorption. At the PANI^0^ state, the nanowire-based plasmonic resonance mainly produces the narrow and strong absorption. On the contrary, in the case of PANI^2+^, the increased imaginary part of the refractive index results in strong absorptive conditions in the planar resonator, whereas the nanowire-based plasmonic resonance is weakened. Therefore, in the PANI^2+^ state, the mainly contributing resonance mode is planar resonance based on the ultrathin resonant mode. Each resonating mode exhibits selective and broad absorption for resonators 1 and 2 (Figure S1). In the reduced state (PANI^0^), the color variation is more sensitive to changes in structural parameters, while in the oxidized state, they become less sensitive. In this context, we will discuss the design parameters and their effects in terms of chromaticity. We note that the resonance structure involves superposed diverse resonant modes of the waveguiding in nanowire, plasmonic modes (i.e., SPP), and other minor resonant modes (e.g., localized surface plasmon). For instance, Si nanowires independently generate a robust HE^11^ waveguiding mode [37], a finding validated through additional simulations (Figure S2). The intensity of this mode is altered based on the deposition of the Au layer and the coating of the PANI layer. Due to the superposition of diverse resonant mechanism, isolating the individual contribution of each mode proves challenging [38]. Nevertheless, we have observed a positive impact, contributing to the strengthening of strong and narrow resonance modes, resulting in distinct coloration.

### 2.2 Optical calculation for enhanced chromaticity in ENP design

**Figure 2:**
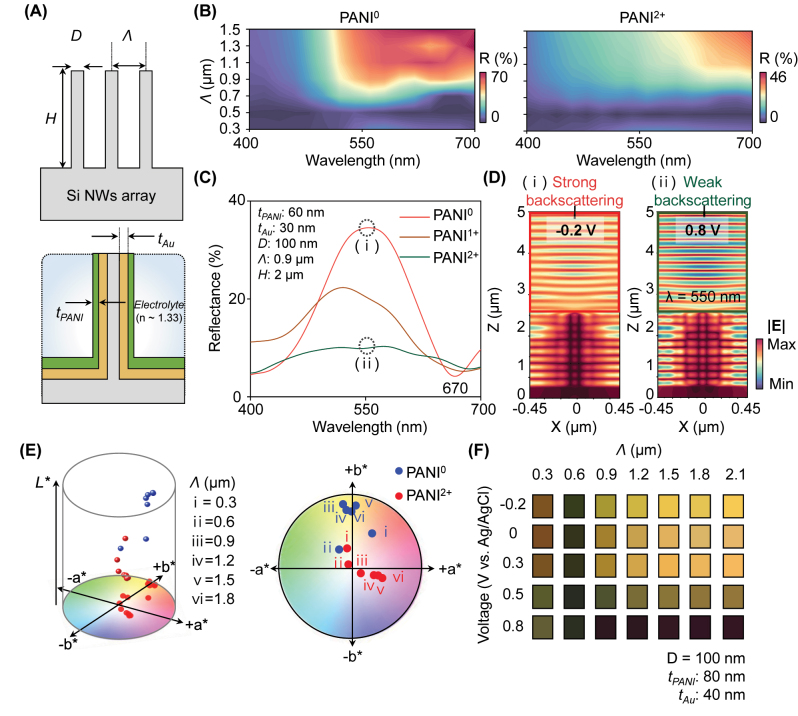
Structural characterization and chromatic analysis of ENP. (A) Schematic illustration of SMCF with geometrical parameters as follows: *D* = 100 nm, *H* = 2 μm, *t*_*Au*_ = 30 nm, and *t*_*PANI*_ = 60 nm. Λ is swept from 0.3 μm to 1.5 μm. (B) Reflectance contours with the redox states of PANI^0^ and PANI^2+^. (C) Reflectance spectra of ENP corresponding to each redox state of PANI. (D) Electric fields of single ENP simulated under the condition of points at (i) and (ii) from (C). (E) Three-dimensional CIE LAB coordinates, which show reflection color of each condition with varying Λ (left) and two-dimensional view (right) from the simulated result. Red and blue dots represent the redox state of PANI^0^ and PANI^2+^, respectively. (F) Color palette of simulated reflection color. At each voltage (vs. Ag/AgCl), the measured complex refractive indices were used.

The cross-sectional schematic of the device is presented in Figure 2A. Si nanowire arrays were selected as templates for ENPs, taking into account the cost-efficiency and productivity associated with Si-based fabrication processes (see Methods and Figure S3). As described in the schematic, its dimension can be expressed with diameter (*D*), period (Λ), and height (*H*). We note that each referred parameter influences the reflectance spectrum. Therefore, we have calculated the tendency (Figure S4). Based on this template, the Au layer as a conducting and reflecting layer is deposited with the thickness of *t*_*Au*_, and sequentially, the PANI layer is coated by the electrodeposition method with *t*_*PANI*_. Also, we considered the refractive index of the background area as *n* = 1.33. Figure 2B shows reflectance contours of ENPs with the structural parameters of *D* = 100 nm, *H* = 2 μm, *t*_*Au*_ = 30 nm, and *t*_*PANI*_ = 60 nm. Λ is swept from 0.3 μm to 1.5 μm. To compare the optical responses of ENPs corresponding to the redox state of the PANI layer, we utilized the complex refractive indices of PANI^0^ and PANI^2+^. We note that, in response to period variations, the resonance wavelength shifts; however, in the range over a period of 0.7 μm (Λ > 0.7 μm), the variation level is not significant in reduced state. Concurrently, there is broad absorption due to the involvement of the resonator (in the oxidized state by planar structure). Also, we emphasize that the primarily considered wavelength range, in terms of color information, is from 450 nm to 650 nm. In this range, the spectral response of the color is sensitive, depending on the tristimulus function [39]. In this regard, the dominant reflection intensity variation is revealed with Λ above 0.7 μm by changing the PANI redox state (PANI^0^ and PANI^2+^). Figure 2C provides a detailed representation of the simulated reflectance spectra for ENP with Λ = 0.9 μm. Notably, the reflectance at *λ* = 550 nm decreased from 37 % to 12 %, resulting in a significant change in chromaticity. At points (i) PANI^0^ and (ii) PANI^2+^, we observed the electric field distributions. Figure 2D visualizes these absorption-based optical resonances using the 3D finite-difference time-domain (FDTD) method (see Methods). As discussed in the previous section, at a wavelength of 550 nm with PANI^0^, the dominant absorption occurs at the standing spot of the Au layer on Si NWs, excluding the PANI layer (Figure 1D; left). In contrast, ENP with PANI^2+^ predominantly absorbs incident light at the PANI layer, resulting in strong absorption (Figure 1D; right). The electric field distributions in the boxed areas depict the scattered light in the backward direction. By monitoring the reflected power, the reflection intensity gradually decreases corresponding to the change in redox state from PANI^0^ to PANI^2+^ (Figure S5). This result indicates that strong backscattering is observed in the PANI^0^ state, whereas light is weakly scattered backward in the PANI^2+^ state. For a quantitative comparison of chromatic analysis, we reconstructed the calculated reflectance spectra into the CIELAB color space, as shown in Figure 2E. To investigate the trends in color variation, we controlled the period of the Si NWs for both redox states, namely PANI^0^ and PANI^2+^. As depicted in the 2D view of the CIELAB space, we observed the large color variation in the level of color difference (i.e., Δ*E*) after the configuration of iii (i.e., Λ > 0.9 μm) between two redox states of PANI with change of color information (hue, saturation, and brightness) (Figure S6). This observed trend is depicted in Figure 2F. Also, we note that the strong broadband absorption significantly absorbs visible light across the entire spectrum, resulting in subtle chromatic variation at the oxidized state (Figure S6). Based on the large chromaticity, from the next section, we experimentally realized and characterized the ENPs using these conditions (Λ = 0.6 μm and 0.9 μm).

### 2.3 Electrochemically controllable optical response and coloration

**Figure 3:**
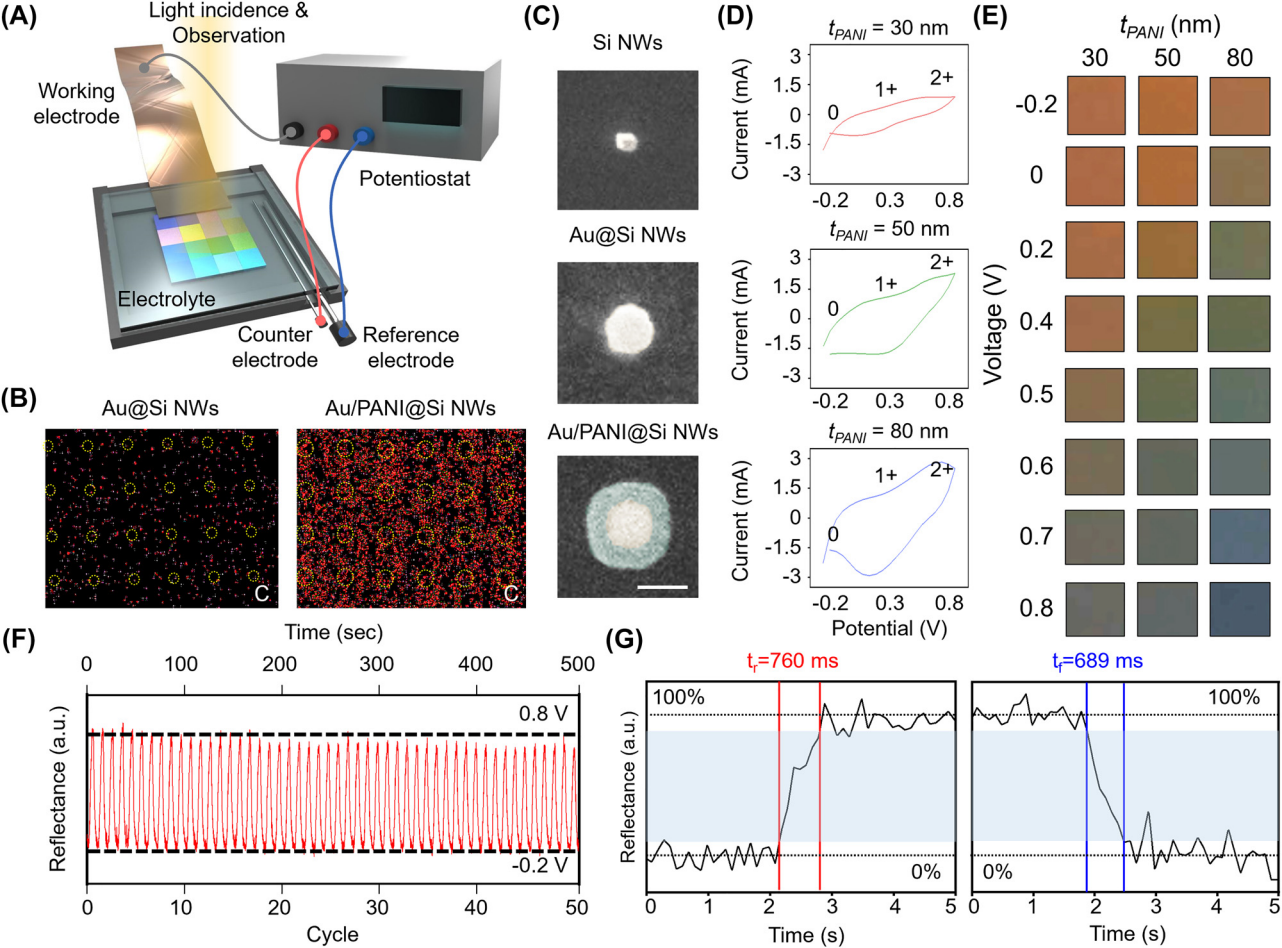
Electrochemical characterization and coloration performance of the ENP. (A) Schematic illustration of optical measurement setup for the ENP. (B) Energy dispersive spectroscopy (EDS) elemental mapping of carbon without (left) and with PANI coating (right). (C) Scanning electron microscope (SEM) image of Si NWs (top), Au@Si NWs (middle), and Au/PANI@Si NWs (bottom). The scale bar is 200 nm. (D) CV curves of the ENPs with applied potential at the varied thickness of the PANI layer (30, 50, and 80 nm from top to bottom). (E) Photograph image corresponding to the applied voltage. (F) Cyclic switching for 50 cycles. The cycle stability by monitoring the reflectance under a voltage range from −0.2 V to 0.8 V. (G) Response time of ENP. The rising and falling times are 760 ms (reduction) and 689 ms (oxidation), respectively.

To examine the electrochemically switchable optical response and coloration performance of the ENP, a customized optical measurement setup was implemented, featuring real-time observation of the chromatic changes in response to the variation of electric potential, as depicted in Figure 3A (see Methods and Figure S7). The energy dispersion spectroscopy (EDS) was employed to scrutinize the spatial distribution of carbon, a constituent of the PANI framework. In Figure 3B, the elemental mapping images elucidate discernible alterations, prominently in the carbon (C). The red points signify the presence of the carbon element, whereas the yellow dashed lines delineate the precise physical localization of the nanowires. It is noteworthy that the component ratios of other elements, e.g., Au, N, Si, remain relatively constant (Figure S8). Furthermore, a systematic demonstration for uniform deposition of each layer is provided by depicting the gradual increase in nanowire thickness from Si NW (top) to Au@Si NWs (middle) and Au/PANI@Si NWs (bottom) through scanning electron microscopy (SEM) images in Figure 3C. To explore the sensitivity of color conversion in ENP to structural parameters, we fabricated ENP samples featuring three distinct PANI layer thicknesses on each Au@Si NWs substrate. The thickness variations, denoted as *t*_*PANI*_, were systematically achieved through a stepwise increment in the number of voltage cycles during the coating process, resulting in *t*_*PANI*_ = 30, 50, and 80 nm for each respective sample (Figure S9). Subsequently, the electrical and optical properties were analyzed based on the complex refractive index of PANI for each state (Figure S10). Figure 3D shows the cyclic voltammetry (CV) curves for distinct PANI thicknesses across the potential span from −0.2 V to 0.8 V. The data indicate a proportional augmentation in the CV curve areas with escalating PANI thickness. This correlation signifies an enhanced electrochemical response corresponding to increased PANI deposition. As described, cyclic voltammetry (CV) curves exhibit distinct optical hysteresis behavior with a varying window size corresponding to the thickness of PANI. This phenomenon may be attributed to several additional geometrical factors. These factors include different thicknesses due to additional effects of their ligands and nonuniform coatings of thinner PANI shells [5]. Quantitative analysis involving the calculation of color difference and color gamut for each condition, derived from the captured images, substantiated that the maximal color variation occurred at *t*_*PANI*_ = 80 nm (Figure S11). This empirical evidence underscores the critical impact of PANI thickness on the observed color changes, indicative of optimized electrochemical performance. Moreover, the electrochemical robustness of the ENP is evident, as it maintains its redox state (oxidation potential) effectively over 50 cycles of voltage modulation, as depicted in Figure 3F. The optically resonant structure, integrated with the active material, achieves rapid activation in just 760 ms (reduction) and efficiently reverts to its initial state within 689 ms during the deactivation process (oxidation), as shown in Figure 3G. These results emphasize the swift and consistent response of the ENP.

### 2.4 Experimental fabrication and optical information encryption

**Figure 4:**
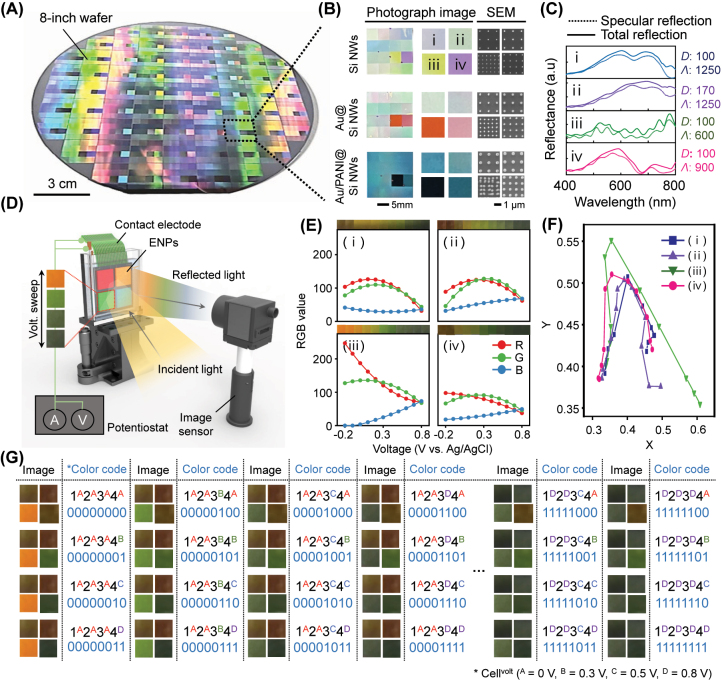
Experimental fabrication of ENPs array and optical information encryption device. (A) Photo image of fabricated 8-inch wafer scale ENPs array. (B) Closed view of ENPs array with photographs and scanning electron microscope (SEM) image. The unit cells of (i), (ii), (iii), and (iv) indicate different design parameters ((i) = *D*170 and Λ1250; (ii) = *D*100 and Λ1250; (iii) = *D*100 and Λ900; (iv) = *D*100 and Λ600, each number represents a dimension in nanometers (nm)). (C) Simulated reflectance of four-unit cells. (D) Schematic of ENPs array with an angular sensitive property. (E) The color palette of four-unit cells corresponding to the different incident angles and voltages. (F) Code chart of the color patterns under four different voltages (0, 0.3, 0.5, and 0.8 V) and incident angles (0°, 20°, 40°, and 60°) on combined unit pixels.

Figure 4A displays an 8-inch scale fabrication of a Si nanowire array. To achieve this large-area fabrication, we employed KrF scanner lithography (Nikon Inc., KrF scanner S203-B) on an 8-inch wafer (see Methods). Figure 4B presents the complete patterns, including 16 segmented regions where the nanopixels are fabricated with various diameters, period, and arrangement (for detailed information about each region, see Figure S12). We deposited Au thin film on these fabricated Si nanowire arrays, sequentially, coated with PANI via electrodeposition. Especially, in this section, we characterized representative four pixels with dimensions as follow: *D* = 100 nm and Λ = 1250 nm for (i); *D* = 100 nm and Λ = 1250 nm for (ii); *D* = 100 nm and Λ = 600 nm for (iii); *D* = 100 nm and Λ = 900 nm for (iv). SEM imaging confirmed the nanopixels fabricated with structural dimensions noted above. Figure 4C displays the simulated reflectance for four representative pixels with the reduced state of PANI, showing both specular and total reflectance spectra. It is noteworthy that the disparity between specular and total reflectance spectra exhibits intensity variation, primarily indicating nanowire-based plasmonic resonance. Conversely, in the oxidized state of PANI, the difference is relatively small, with the dominance of the ultrathin resonant mode (Figure S13).

Taking advantage of the significant contrast observed in the varying redox states of PANI, we conducted multi-level optical information encryption using different voltage levels. As depicted in Figure 4D, our measurement setup involves two crucial parts, one for electrical control and another for optical measurement (Figure S14). Figure 4E presents the experimental results with photographic images. The RGB values derived from the captured images represent substantial and distinct variations corresponding to the applied voltage and pixel (Figure S15). As shown in Figure 4F, the chromatic information was expressed in CIE coordinates, demonstrating various points covering a broad area in the red, green, and blue regions. Finally, we configured the unit cell with four pixels, and then each region was selectively addressed with different voltages: 0 V, 0.3 V, 0.5 V, and 0.8 V. By applying voltage to the target pixel, we generated 2 bits per pixel. By combining these bits, we encoded a total of 8 bits of chromatic information per unit cell for optical reading (Figure 4G).

### 2.5 Angular dependency of ENP

**Figure 5:**
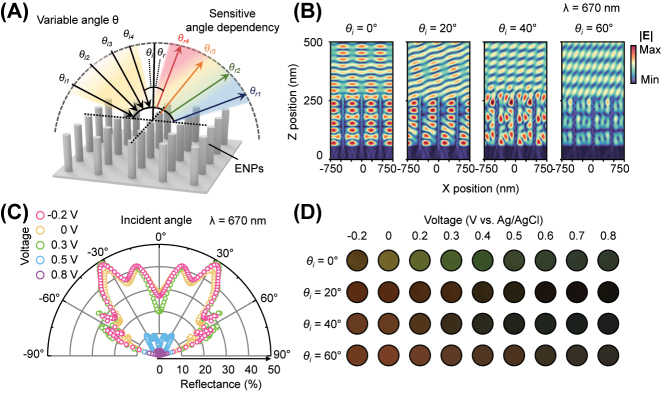
Angle dependency of ENP. (A) Schematic of ENP with angular response. The arrows represent the reflection direction by angular factor (*θ*_*r*_). (B) Electric field distribution of ENP with the dimension of *D* = 100 nm, and Λ = 900 nm corresponding to different incident angles (*θ*_*i*_). (C) Calculated reflectance at *λ* = 670 nm corresponding to different incident angles (*θ*_*i*_) and applied voltage (V vs. Ag/AgCl). (D) Photograph image with different incident angles (*θ*_*i*_) and applied voltage (V vs. Ag/AgCl).

One of the key advantages of the electrochromic nanoxpixels formed with a high aspect ratio renders angular-dependent color, acting as an additional degree of color tuning. Figure 5A illustrates the angular response of ENP. Figure 5B shows an electric field distribution corresponding to the angle of incidence with reduced state of PANI (PANI^0^). Depending on the incident angle *θ*_*i*_, the reflectance intensity fluctuates significantly due to the sensitive responsiveness of waveguide mode. We observed that when light is incident at small angles relative to the nanowire axis, it mainly couples with nanowire-based plasmonic resonance [40]. However, this mode is absorbed as it travels through the wire. On the other hand, at larger angles of incidence, the coupling to the guided mode weakens, and the incident light instead excites ultrathin resonances, which become the primary absorption mechanism. This tendency is illustrated in Figure 5C. However, under 0.8 V (vs. Ag/AgCl), the ENP demonstrates less sensitive modulation in reflectance because the dominant resonance mode is the ultrathin resonance rather than the nanowire-based plasmonic resonance. This trend is evident in the chromatic variation depicted in Figure 5D. The color palette illustrates how color changes correspond to the incident angle and applied voltage. As described, the color variation is more pronounced in the reduced state (at relatively low voltage) than in the oxidized states (at relatively high voltage). We note that this sensitive angular property could extend the capacity for optical data encryption by providing an expanded amount of information from a single cell. We note that, due to the optical hysteresis property of PANI, during the overall demonstration, we applied the encryption state in the forward direction (from zero voltage to the target potential).

## 3 Conclusions

In summary, this work demonstrated an electrically controllable nanopixel array based on the redox reaction of PANI, which offers a promising solution for enhanced chromaticity from a low complex refractive index. The transition of PANI from a lossy to a dielectric state is central to our approach. We designed the photonic structure to be responsive to the presence of the extinction coefficient, utilizing the sensitivity of the SPP-based waveguiding mode to the complex refractive index of the surrounding medium integrated with planar broadband absorber. Moreover, our device achieves a broad chromatic range spanning red, green, and blue colors while operating efficiently on a sub-1-volt input voltage, in line with the conventional CMOS voltage standard. This work extends beyond the theoretical framework to practical applications, particularly in optical encryption. We experimentally demonstrate an array of hybrid nanowires with diverse structural parameters on an 8-inch wafer scale. The optical encryption system employs encoding and decoding methodologies that encompass varied techniques, notably the utilization of a polarization-tunable color-generator [41] and the modulation control of the electro-optic modulator (EOM) within the laser [42], [43]. Leveraging enhanced chromaticity, each unit cell with four different pixels generates 8 bits of optical information per unit cell. By harnessing the high sensitivity to the angular dependency of the waveguiding mode, we further expand the information capacity to an impressive 10 bits per unit cell. Notably, our approach surpasses prior studies where the maximum achievable encryption was limited to 7 bits. Lastly, as a future outlook for our proposed concept, it will be possible to enhance color variation levels (purity and hue value), allowing for the storage of even more diverse information. Essentially, the prerequisite for increasing color purity is presenting a narrowband reflection. The proposed structure features an array of nanowires, and the resonant wavelength can be adjusted based on the configuration changes, especially period. Therefore, we believe that by forming diverse nanowire domains with different periods and implementing multiple resonating structures to create resonance at two wavelengths within the visible spectrum, we can achieve a high level of color purity [44]. Looking ahead, we envision further advancements aimed at exploring coupling effects based on combining sensitive responses for the variation of PANI to enlarge the optical responses for complex light modulation, i.e., both amplitude and phase, in visible wavelength range.

## 4 Methods

### 4.1 Optical calculation

The optical spectra were calculated through the utilization of commercially available software (DiffractMOD, RSoft; USA) employing the rigorous coupled wave analysis (RCWA) method. To ensure numerical stability and precision in all simulation outcomes, the ninth diffraction order and a grid size of 1 nm were considered. For absorption and electric field simulations, the finite-difference time domain (FDTD) method was applied, with perfectly matched layer (PML) domains used in both horizontal and vertical directions. In order to attain accurate calculation results, the material dispersions and extinction coefficients were considered. The complex refractive indices of the PANI were measured by ellipsometer. Also, the refractive index of Si and Au was obtained from the literature [45], [46]. The background refractive index was configured to 1.33, as it corresponds to the environment containing mainly deionized water.

### 4.2 ENP fabrication

Initially, a Si substrate underwent thermal annealing to generate a silicon dioxide (SiO_2_) layer approximately 200 nm thick. Subsequently, square pattern of SiO_2_ nanodisk were fabricated, with various diameters ranging from 200 to 250 nm with 10 nm increments, using KrF scanner lithography (Nikon Inc., KrF scanner S203-B). Reactive ion etching (RIE, Oxford Plasmalab 133) was employed to etch the Si substrate to a depth of about 2 μm. Hydrofluoric acid (HF) treatment was then applied to eliminate SiO_2_ residues, and the etched wafer underwent precise thermal oxidation to reduce the nanowire diameters. Lastly, the process concluded with an HF wet etching step to remove any remaining SiO_2_ residues. After the formation of Si nanowire arrays, an Au layer (∼40 nm thick) was deposited using a magnetron radio-frequency sputtering system (DDHT-LSH2, Daedong High Tech., Korea) under a high vacuum (≈10^−6^ Torr). The PANI was electrodeposited employing a potentiostat (PARSTAT4000A, AMETEK, USA) within a potential window spanning from −0.2 V to 0.8 V (scan rate: 0.05 V/s) versus Ag/AgCl reference electrode, subsequently. The electrolyte solution to grow the PANI layer comprised a mixture of 50 mmol aniline monomer and 2 mol HNO_3_.

### 4.3 Optical measurement and characterization

The dynamic color modulation of the ENP was executed through potentiostat, maintaining a potential window from −0.2 V to 0.8 V versus Ag/AgCl, employing an electrolyte solution composed of 0.5 mol NaCl dissolved in a 10 mmol HCl base. Electrically responsive optical characteristics were quantified in customized cartage equipped with a UV-VIS-NIR spectrometer (LAMBDA 950, Perkin Elmer, USA) at a normal incidence. A tungsten–halogen lamp served as the light source. SEM images of the ENPs were obtained using an FE-SEM (Quanta 200 FEG, FEI Corp.) at an accelerating voltage of 20.00 kV.

### 4.4 Optical information encryption

Information encryption of ENPs was implemented under same conditions at dynamic color modulation. It was executed through potentiostat while maintaining a potential window from −0.2 V to 0.8 V relative to Ag/AgCl using an electrolyte solution composed of 0.5 mol NaCl dissolved in a 10 mmol HCl base. To secure the amount of light, the mounted LED light (MCWHL6, Thorlabs, USA) was incident in the normal direction and the electrochromic characteristics were photographed through the camera. In addition, in order to measure the angle dependency of ENPs, a rotation stage (PR01, Thorlabs, USA) was added under the chamber to observe color changes according to angles.

## Supplementary Material

Supplementary Material Details
