# Supplementary material for: Electrochromic nanopixels with optical duality for optical encryption applications
Source: Nanophotonics. 2024 Jan 12;13(7):1119–29. doi: 10.1515/nanoph-2023-0737 (PMC11501882; doi:10.1515/nanoph-2023-0737)
Supplement: Supplementary file 1 — Supplementary Material Details [file j_nanoph-2023-0737_suppl_001.pdf]

Supporting Information for

Electrochromic nanopixels with optical duality for optical encryption applications

Joo Hwan Ko, Ji-Eun Yeo, Hyo Eun Jeong, Dong Eun Yoo, Dong Wook Lee, Yeon-Wha Oh, Sanghee Jung, Il-Seok Kang*, Hyeon-Ho Jeong*, Young Min Song*

Corresponding authors: National Nanofab Center, Korea Advanced Institute of Science and Technology, 291 Daehak-ro, Yuseong-gu, Daejeon 34141, Republic of Korea; iskang@nnfc.re.kr (Il-Seok Kang); and School of Electrical Engineering and Computer Science and Department of Semiconductor Engineering, Gwangju Institute of Science and Technolo-gy, Gwangju 61005, Republic of Korea; jeong323@gist.ac.kr; https://orcid.org/0000-0002-7029-9592 (Hyeon-Ho Jeong); and School of Electrical Engineering and Computer Science, Department of Semiconductor Engineering, and Artificial Intelligence (AI) Graduate School, Gwangju Institute of Science and Technology (GIST), Cheomdangwagi-ro 123, Buk-gu, Gwangju 61005, Republic of Korea; ymsong@gist.ac.kr; https://orcid.org/0000-0002-4473-6883 (Young Min Song)

Joo Hwan Ko, Ji-Eun Yeo, and Hyo Eun Jeong: School of Electrical Engineering and Computer Science, Gwangju Institute of Science and Technology (GIST), Cheomdangwagi-ro 123, Buk-gu, Gwangju 61005, Republic of Korea;

Dong Eun Yoo, Dong Wook Lee, Yeon-Wha Oh, Sanghee Jung: Korea Electronics Technology Institute, Ballyong-ro 111, Deokjin-gu, Jeonju 54853, Jeollabuk-do, Republic of Korea

S1. Reflectance comparison between nanowire array and planar resonator

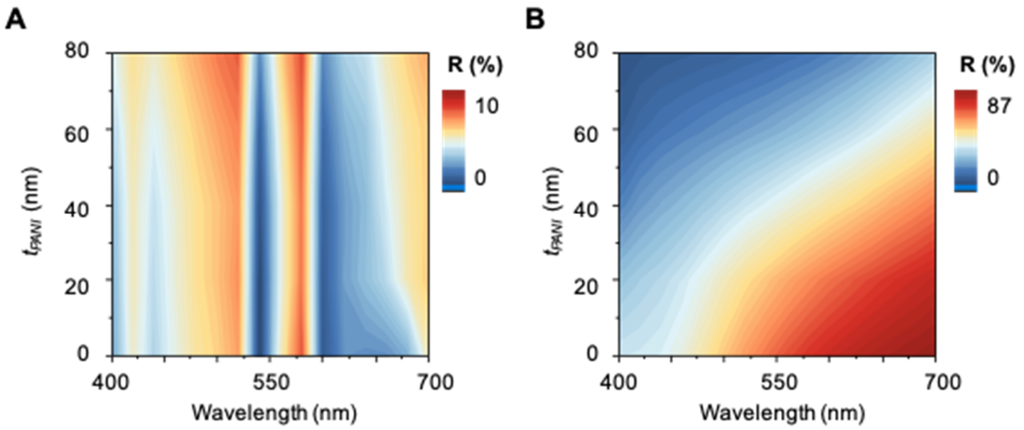

Figure S1. (A) Reflectance contour of nanowire array without planar resonator layer with sweeping the thickness of PANI with the structural parameters of t_{Au} = 40 nm, diameter of Si = 100 nm, and the reduced state ($PANI^0$). **(B)** Reflectance contour of planar resonator without nanowire array layer with sweeping the thickness of PANI with the structural parameters of t_{Au} = 40 nm, diameter of Si = 100 nm, and the oxidized state ($PANI^{2+}$).

S2. Absorption calculation at different structure

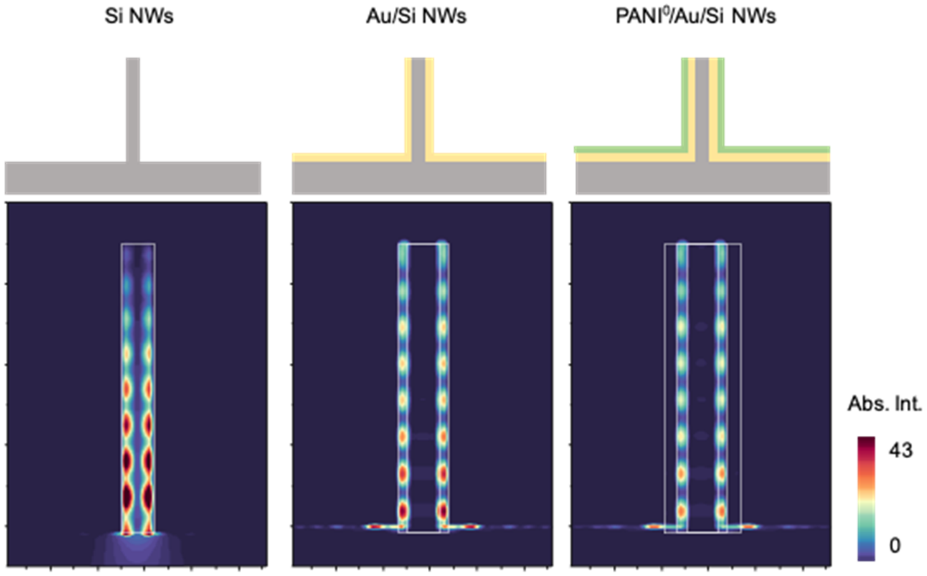

Figure S2, Schematic of the calculation configuration of Si nanowire, Au coated Si nanowire, and Au/PANI coated Si nanowire. Based on the configuration, the calculation results show the absorption power with different range and intensity. It is noteworthy that Si nanowire shows strong waveguiding mode and by adding the coating layer the waveguide intensity is strongly influenced. The simulation was performed at the wavelength of 670 nm.

S3. Fabrication step

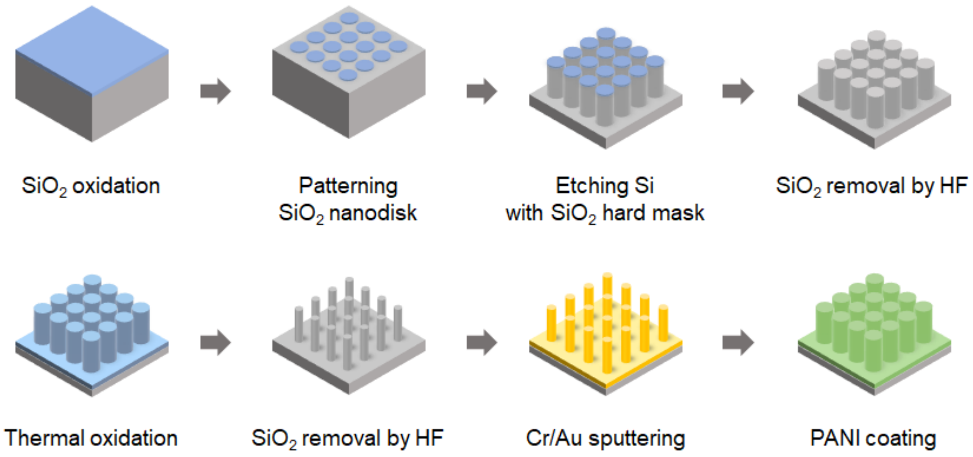

Figure S3. Schematic illustration of the fabrication process of electrochromic nanopixels (ENPs).

S4. Design rules of ENP

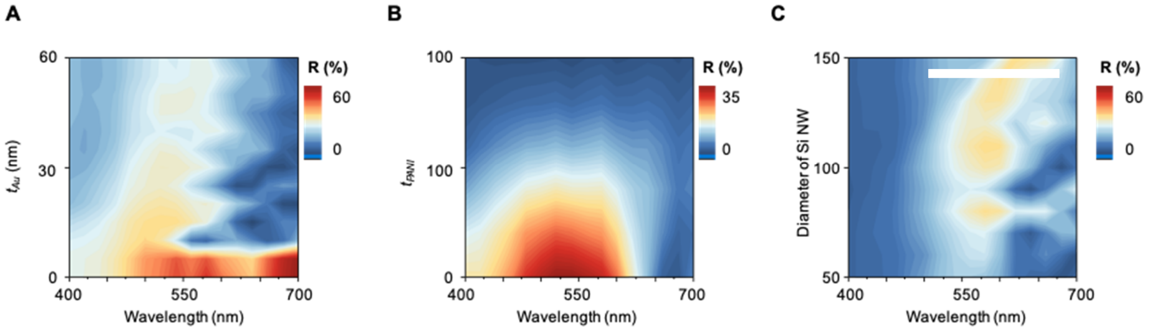

Figure S4. (A) Reflectance contour with sweeping the thickness of Au with the structural parameters of t_{PANI} = 80 nm, diameter of Si = 100 nm, and the reduced state ($PANI^0$). (B) Reflectance contour with sweeping the thickness of PANI with the structural parameters of t_{Au} = 40 nm, diameter of Si = 100 nm, and the oxidized state ($PANI^{2+}$). (C) Reflectance contour with sweeping the diameter of Si with the structural parameters of t_{Au} = 40 nm, t_{PANI} = 80 nm, and the reduced state ($PANI^0$).

S5. ENP reflectance power monitoring

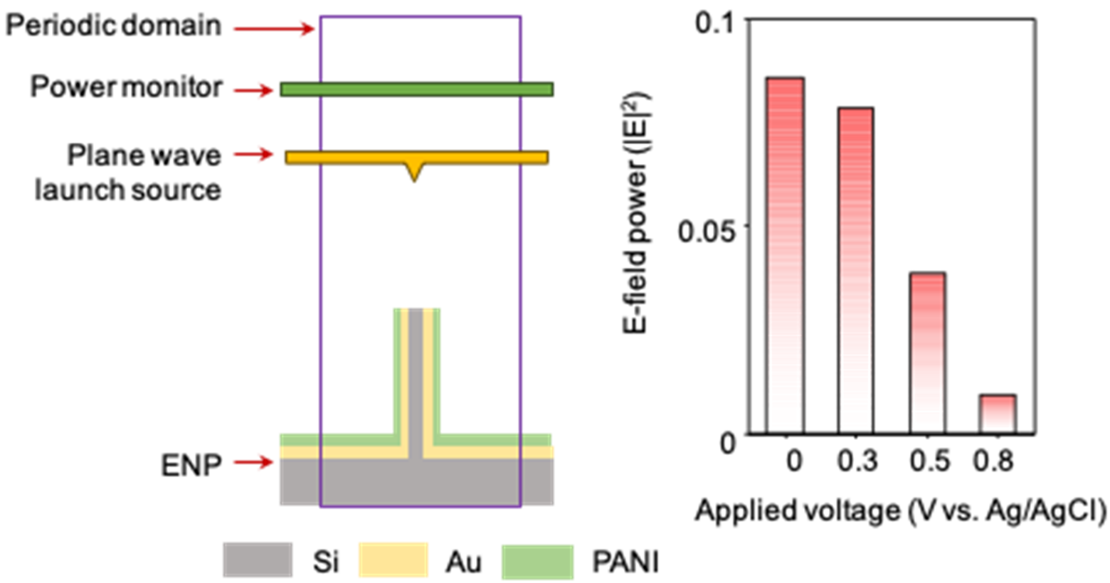

Figure S5. Schematic of the calculation environment for determining the reflection power of ENP based on the change in the redox state of PANI (left). The calculation results show the electric field power corresponding to the variation in the complex refractive index of PANI under different applied voltages (right). The simulation was performed at the wavelength of 550 nm.

S6. Color information of PANI

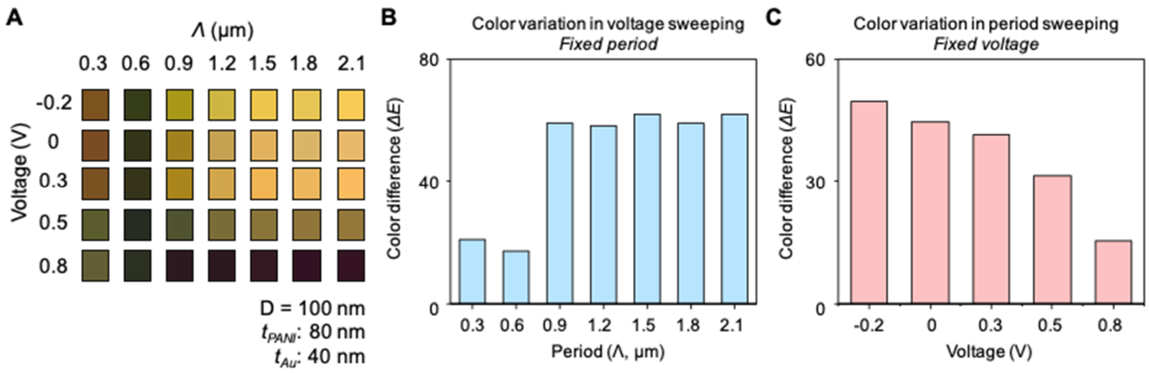

Figure S6. (A) Color palette of simulated reflection color. At each voltage (vs. Ag/AgCl), the measured complex refractive indices were used. (B) Color difference (ΔE) representing color variation in voltage sweeping for each fixed period. (C) Color difference (ΔE) representing color variation in period sweeping for each fixed voltage (redox state of PANI).

S7. Optical measurement setup

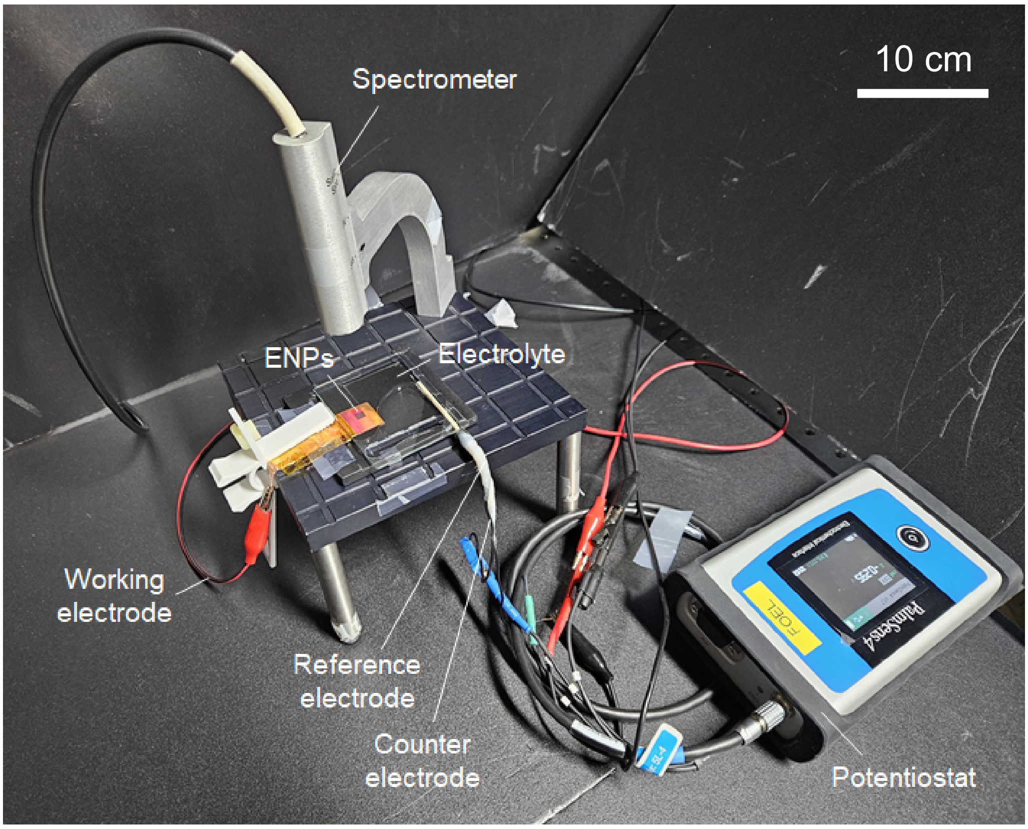

Figure S7. The image of optical measurement setup for ENPs by monitoring the color dynamics via spectrometer, observed under a voltage range from -0.2 V to 0.8 V.

S8. EDS spectrum and elemental mapping

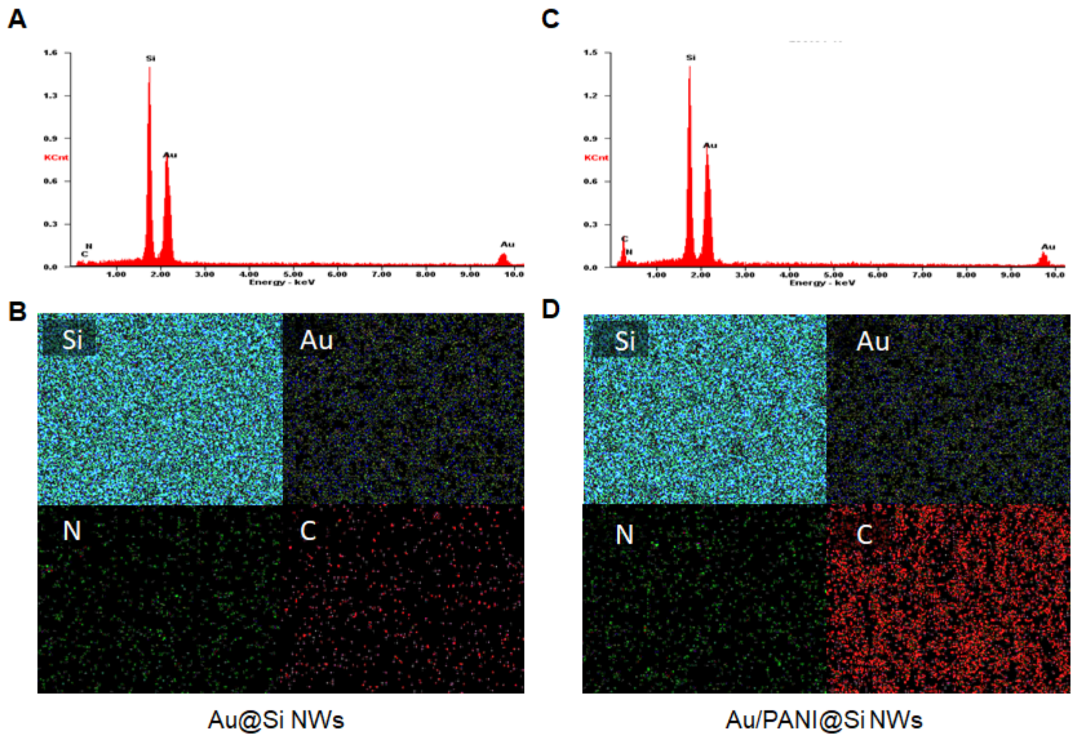

Figure S8. (A-B) EDS spectrum (top) and elemental maps (bottom) for four elements (Si, Au, N, and C) on Au@Si NWs surface. (C-D) EDS spectrum (top) and elemental maps (bottom) for Si, Au, N, and C on Au/PANI@Si NWs surface.

S9. Electrochemical deposition of PANI on Au@Si NWs

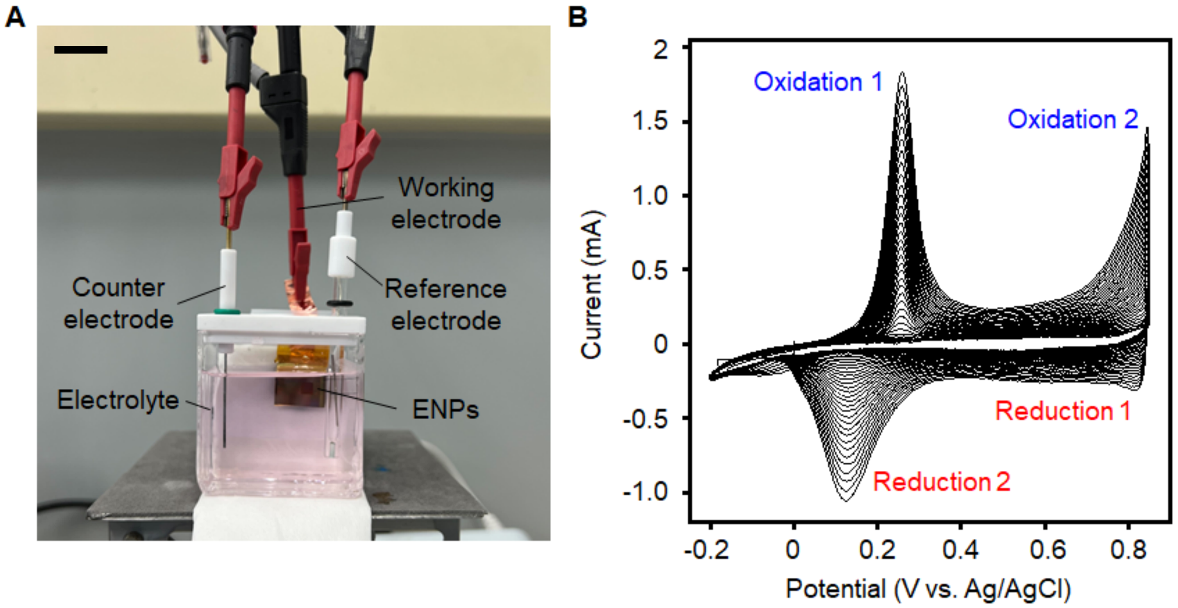

Figure S9. (A) Photographs of PANI deposition setup. The ENPs were directly used as working electrode contacted by Cu tape. Ag/AgCl and Pt were used as reference and counter electrodes, respectively. The scale bar is 2 cm. (B) Cyclic voltammetry (CV) analysis illustrating PANI growth in relation to applied voltage.

S10. Spectroscopic ellipsometry data of PANI

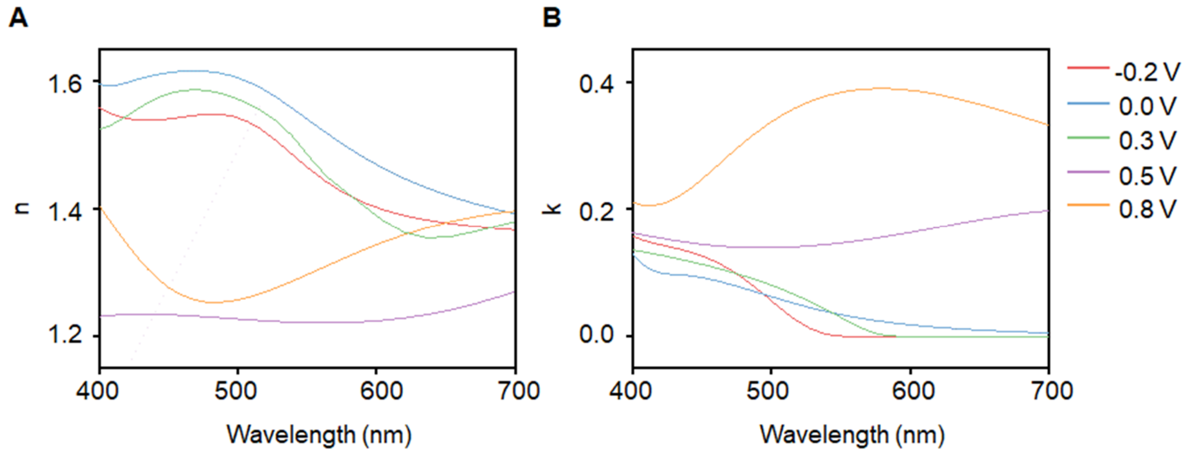

Figure S10. (A) Real part and B) imaginary part of the complex refractive index of PANI by measured by ellipsometry.

S11. Color difference and gamut

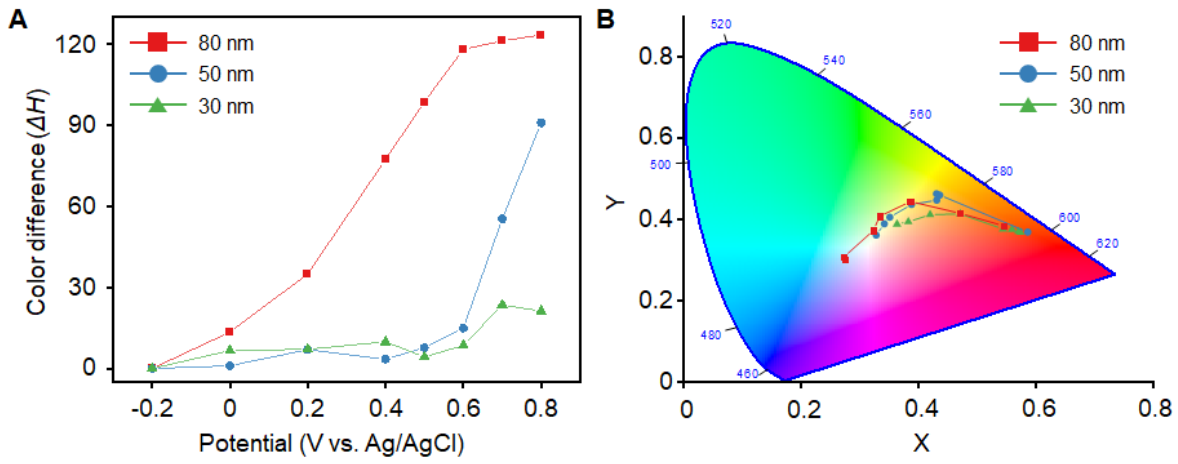

Figure S11. (A) Measured color difference and (B) corresponding color gamut of ENPs with applied potential, showcasing variations at different PANI thicknesses (30, 50, and 80 nm). Increased PANI thickness correlates with higher color difference and a broader color gamut.

S12. Cell dimension

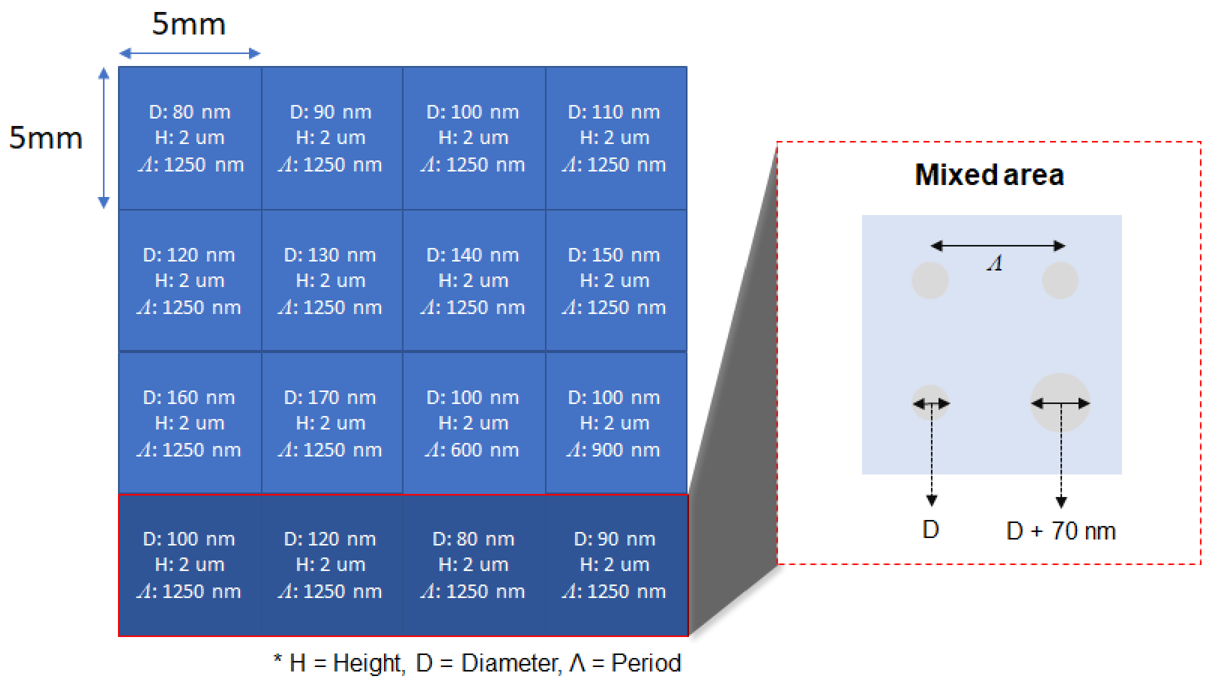

Figure S12. The cell information of Si NWs. *H*, *D* and *Λ* represent height, diameter, and period, respectively.

S13. Calculated reflectance of total and specular reflection

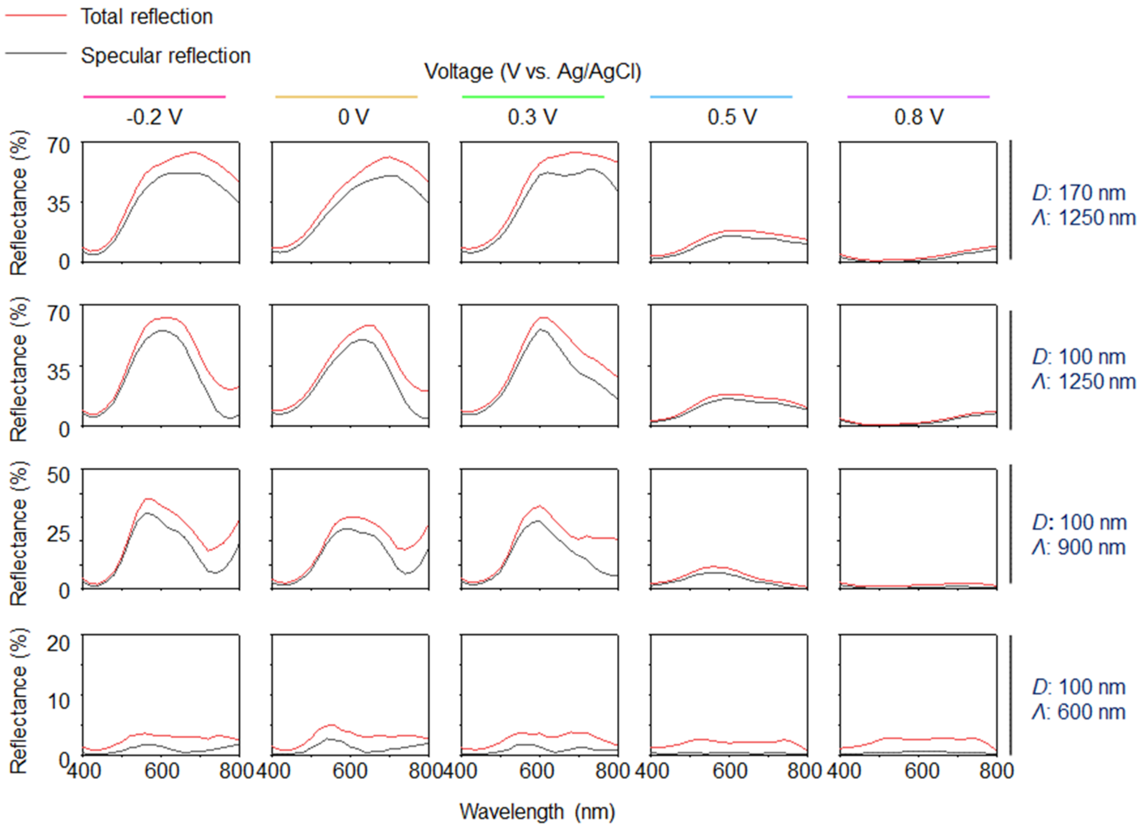

Figure S13. Calculated specular reflectance and total reflectance corresponding to different dimension and applying voltage.

S14. Demonstration setup

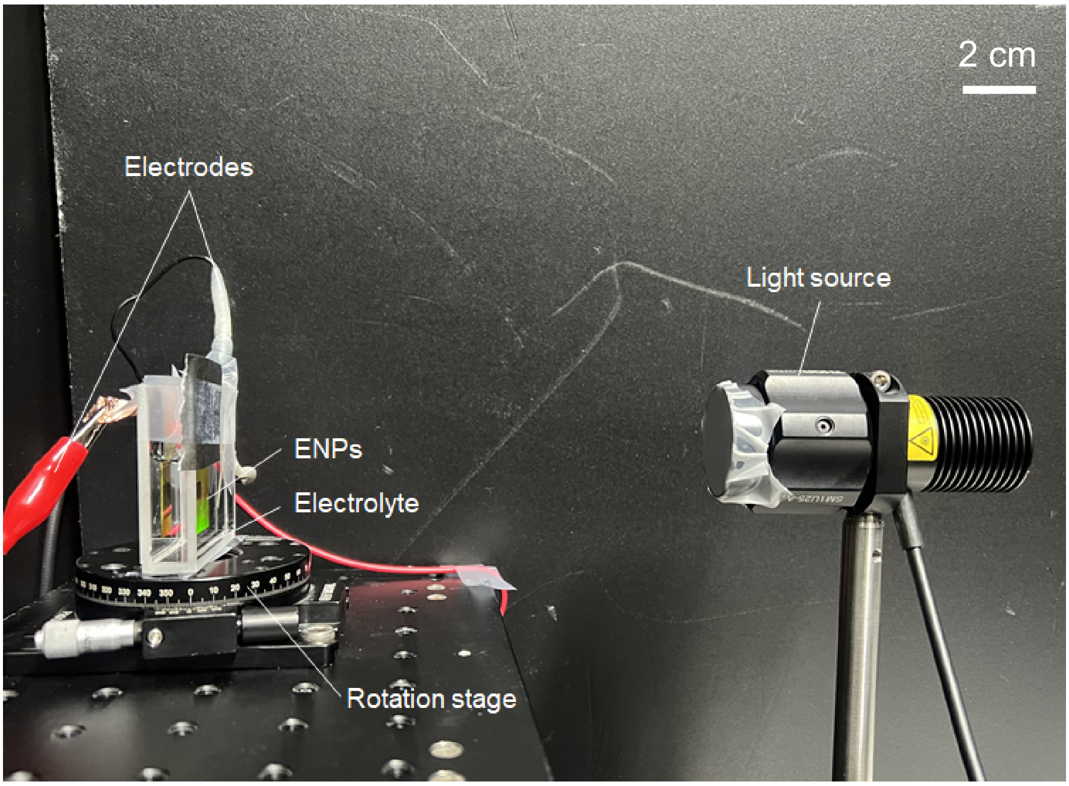

Figure S14. The image of optical demonstration setup. For optical information encryption at a voltage range from -0.2 V to 0.8 V, additional white light was used to enter the sample to secure the amount of light. In order to measure the reflected light, a camera was placed in a direction perpendicular to the sample to photograph the color change. In addition, the angle of reflected light was adjusted using the rotation stage to confirm the angle dependence of the ENPs.

S15. Color palette of four-unit cells

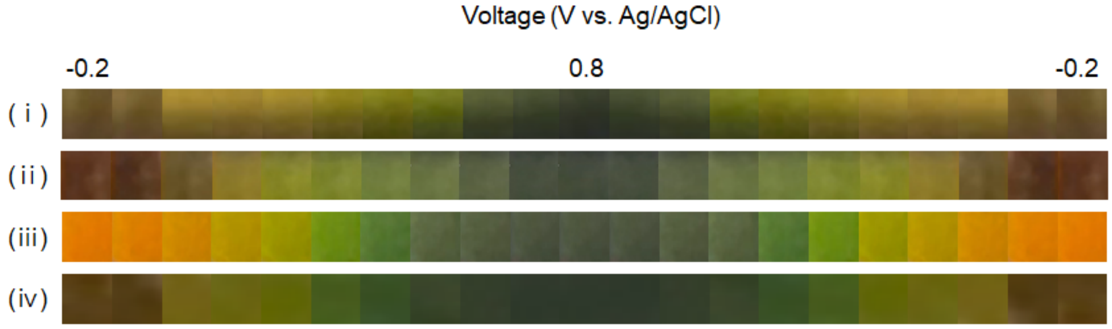

Figure S15. The color dynamics of four-unit cells ((i) *D*: 170 nm and *Λ*: 1250 nm, (ii) *D*: 100 nm and *Λ*: 1250 nm, (iii) *D*: 100 nm and *Λ*: 900 nm, (iv) *D*: 100 nm and *Λ*: 600 nm) corresponding to different voltage.
